# An Optimized Bioassay System for the Striped Flea Beetle, *Phyllotreta striolata*

**DOI:** 10.3390/insects16050510

**Published:** 2025-05-10

**Authors:** Liyan Yao, Xinhua Pu, Yuanlin Wu, Ke Zhang, Alexander Berestetskiy, Qiongbo Hu, Qunfang Weng

**Affiliations:** 1 National Key Lab of Green Pesticide, College of Plant Protection, South China Agricultural University, Guangzhou 510642, China; 13727234833@163.com (L.Y.); puxh@stu.scau.edu.cn (X.P.); lynn500915@163.com (Y.W.); zhangke@scau.edu.cn (K.Z.); hqbscau@scau.edu.cn (Q.H.); 2 All-Russian Research Institute of Plant Protection, Saint-Petersburg 196608, Russia; aberestetskiy@vizr.spb.ru

**Keywords:** entomopathogenic fungi, larvae rearing, bioassay system optimization, germination rate, sulforaphane, host plant

## Abstract

The striped flea beetle (SFB), *Phyllotreta striolata*, is a major pest of Brassicaceae crops, causing substantial yield losses worldwide. Effective biocontrol strategies, particularly the development of mycoinsecticides, require the identification of virulent entomopathogenic fungi (EPF) and the establishment of reliable bioassay systems. However, establishing reliable bioassay systems for SFB has been particularly challenging, especially for larval stages due to their recalcitrant rearing requirements. This study aimed to establish a standardized bioassay protocol to evaluate EPF efficacy against SFB. A specialized larval collection apparatus was developed, and the virulence of three EPF strains (*Beauveria bassiana* BbPs01, *Metarhizium robertii* MrCb01, and *Cordyceps javanica* IjH6102) was assessed against both adult and larval stages using a radish slice-based rearing system. Intriguingly, BbPs01 and MrCb01 exhibited significantly higher LT_50_ values in larvae than in adults, contrary to the typical pattern of greater larval susceptibility observed in most insect systems. We hypothesized that isothiocyanate—specifically sulforaphane, a compound abundant in radish tissues—exerts fungistatic effects that impair fungal growth and virulence. Follow-up experiments confirmed that radish-derived sulforaphane inhibited fungal activity. Through alternative host plant screening, Chinese flowering cabbage (*Brassica campestris* L. ssp. *chinensis* var. *utilis*) was identified as an optimal larval diet that minimally interferes with EPF bioactivity, enabling reliable virulence assessments. This study presents critical methodological advancements for SFB biocontrol research, providing a robust framework for standardized larval bioassay and novel insights into plant secondary metabolite interactions with entomopathogens. The optimized system supports the development of targeted mycoinsecticides and contributes to a deeper understanding of tri-trophic interactions in crucifer pest management.

## Simple Summary

The striped flea beetle (*Phyllotreta striolata*) is a destructive pest of cabbage and other cruciferous vegetables, causing significant crop losses. Biological control using entomopathogenic fungi offers a promising alternative to chemical pesticides, but testing their effectiveness is challenging due to the difficulty of rearing this insect in the laboratory. In this study, we optimized a rearing and testing system for striped flea beetle larvae, discovering that feeding them Chinese flowering cabbage instead of radish reduced unwanted fungal growth and improved test accuracy. Interestingly, we also observed that fungal treatments were more effective against adults than larvae, which is uncommon in most insect systems. Our findings provide a foundation for developing targeted fungal-based biopesticides and contribute to improved pest management strategies for crucifer crops.

## 1. Introduction

The striped flea beetle (SFB), *Phyllotreta striolata* Fabricius (Coleoptera: Halticidae), is an oligophagous pest that infests cruciferous crops worldwide. Both the adults and larvae of SFB cause significant damage to cruciferous vegetables, impacting the entire crop growth cycle. Adult beetles feed on tender shoots, stems, leaves, flowers, and buds, often congregating on the leaf surface and gnawing on plant tissues [1]. Meanwhile, larvae feed on the roots of vegetables in the soil, penetrating the cortex of the main root of crops, which leads to wilting and, in severe cases, plant death [2]. Feeding wounds also facilitate the entry of pathogens, promoting the spread of diseases such as bacterial soft rot and black rot [3,4], which aggravates the occurrence of soil-borne diseases and causes a series of losses. Due to its severe impact on cruciferous vegetable production in southern China, SFB was listed as a class II important pest by Guangdong Province in 2021 [4,5]. The life cycle of SFB includes two stages: aboveground and belowground. Eggs, larvae, and pupae develop in the soil, while the adults inhabit and feed on aboveground plant parts. SFB adults prefer to feed on tender plant stems, buds, leaves, flowers, and fruits, while larvae feed on the roots, causing extensive underground harm and facilitating disease entry [6,7]. Currently, control strategies primarily focus on the adult stage through spraying chemical pesticides [8,9]. However, the species exhibits tolerance to insecticides, which causes large pesticide consumption for higher control efficacy, leading to the problems of residue and product safety. Therefore, there is an urgent need to develop environmentally friendly biocontrol agents as part of a sustainable integrated pest management (IPM) strategy for SFB.

Entomopathogenic fungi (EPF) can directly penetrate the insect cuticle and rapidly proliferate within a wide range of insect hosts, such as Lepidoptera, Coleoptera, Hymenoptera, Hemiptera, and Orthoptera [10]. EPF exhibit unique mechanisms of action by penetrating the cuticle to infect insect hosts, which is similar to the contact killer of chemical pesticides. Notably, EPF can cause epidemics under field conditions, resulting in the natural suppression of pest populations [11,12]. However, the virulence of EPF is influenced by various external and biological factors; for example, pest symbiotic microorganisms can neutralize the virulence of EPF. The leaf beetle *Plagiodera versicolora* relies on its associated microbiota, particularly *Enterobacter* species, to suppress the entomopathogenic fungus *Aspergillus nomius* [13]. *Beauveria bassiana* chrysovirus 2 (BbCV2) infection increased the growth rate, spore production, and biomass of *B. bassiana*, and enhanced its inhibitory ability against plant pathogenic fungi [14]. In addition, the virulence of EPF varies significantly among strains. The study found that *Lecanicillium attenuatum* JL-003 and *B. bassiana* JL-005 strains were more effective than *Lecanicillium longisporum* JL-006 and *Akanthomyces lecanii* JL-007 in controlling *Bemisia tabaci*. Compared with 1st, 2nd, and 3rd instar nymphs, 4th instar nymphs, eggs, and adults of *B. tabaci* were less susceptible to all fungal strains [15]. EPF are soil-dwelling and endophytic fungi that colonize the rhizosphere and contribute to plant defense against insect pests [16,17,18]. This ecological niche and infection pathway make EPF particularly suitable for targeting soil-dwelling pests such as SFB [7], offering promising prospects for sustainable biological control.

However, screening bioactive EPF strains against SFB remains a significant challenge, primarily due to the difficulty in rearing the insect—especially the larval stage. Although laboratory methods for rearing SFB have been reported [19], Nagalingam’s method can be improved to provide more stable feeding conditions and bioassay systems. In practice, we find that the EPF strains tend to exhibit higher activity against SFB adults than larvae, which contradicts the typical pattern observed in EPF bioassays, where larvae are generally more susceptible. We hypothesize that this discrepancy may stem from the larval rearing methods, in which the larvae were fed radish containing rich glucosinolates, which are SFB’s important secondary metabolites playing a key role in defense against pathogens and insects [20,21]. Therefore, optimizing the larval rearing method is critical for establishing a reliable and representative bioassay system.

The purpose of this study is to develop and validate a bioassay system for accurately screening active EPF strains against both larval and adult stages of SFB.

## 2. Materials and Methods

### 2.1. Fungi, Plants, and Insects

The three EPF strains, *Beauveria bassiana* BbPs01, *Metarhizium robertii* MrCb01, and *Cordyceps javanica* IjH6102, which exhibited higher activity against adults of SFB in our previous experiments, were selected for use in this study. The conidia were inoculated from slant cultures onto PDA plates and incubated at 26 °C for 2 weeks. Following incubation, the conidia were collected from the PDA plates and suspended in a 0.02% Tween-80 solution to prepare a stock suspension with a concentration of 1 × 10^8^ spores/mL for further use.

Five cruciferous plants were used to rear SFB: Chinese flowering cabbage (*Brassica campestris* L. ssp. *chinensis* var. *utilis* Tsen et al.), pakchoi (*B. campestris* L. ssp. *chinensis* Makino var. *communis* Tsen et Lee), cabbage (*B. oleracea* L. var. *capitata* L.), Chinese kale (*B. alboglabra* L. H. Bailey), and radish (*Raphanus sativus* L.). Seeds of all plant species were bought from the market (Guangdong Kenong Vegetable Seeds Co. Ltd., Guangzhou, China) and grown in pots in a greenhouse for subsequent applications.

Adults SFB were collected from the experimental farm of South China Agricultural University (Guangzhou, China) and reared with Chinese Flowering Cabbage seedlings in the cages (55 × 55 × 55 cm). They have been maintained in our laboratory for more than 15 generations, establishing a stable laboratory population.

### 2.2. Bioassay of EPF Bioactivity to SFB

Conidia suspensions (1 × 10^8^ spores/mL) were prepared by suspending stock conidia in a 0.02% Tween-80 solution. Adult SFBs were collected from cages using an insect aspirator and anesthetized with carbon dioxide. Then, the anesthetized beetles were the immersed in EPF conidial suspensions for 10 s. After drying, the beetles were transferred into a Petri dish (7 cm) lined with filter paper on the bottom and fed fresh leaves of Chinese flowering cabbage every day. Each treatment group consisted of 20 beetles, and each group was repeated three times. A solution of 0.02% Tween-80 without conidia was used as the control, also with three repeats. The mortality of the infected beetles was observed daily. An insect was considered dead if it did not move when its body (feet or antennae) was touched with a fine brush. The dead beetles were transferred to a Petri dish with high humidity conditions to observe the development of mycosis caused by EPF. The entire experiment was replicated twice.

**Figure 1:**
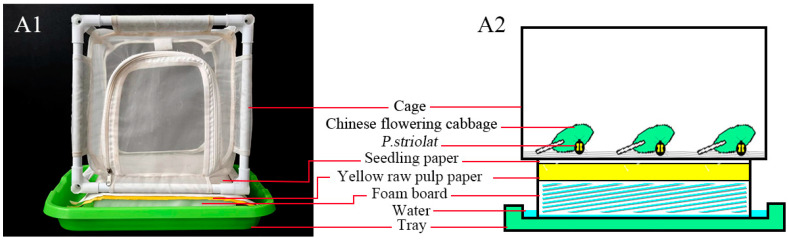
Structure of the SFB adults rearing device with eggs collector. (**A1**) Physical object drawing of the rearing device; (**A2**) Schematic diagram of the rearing device structure: The device consists of, from top to bottom, a piece of seedling-growing paper, three layers of yellow raw pulp paper, a square foam (19 × 19 × 5 cm), and a tray filled with sterile water. Approximately 200 to 300 adults were placed inside the cage, and fresh Chinese flowering cabbage was placed as feed and changed every 2–3 days.

To obtain the larvae for bioassay, an egg-collector was designed (Figure 1) based on Nagalingam et al. [19]. The protocol included three steps: **(1)** *Adult rearing*. Adults were moved from the colony to a cage (20 × 20 × 20 cm) with a 4 mesh plastic net at the bottom. The cage was placed above an oviposition device (Figure 1). From top to bottom, the device consisted of a piece of seedling-growing paper, three layers of yellow raw pulp paper, a square foam (19 × 19 × 5 cm), and a tray filled with sterile water. Fresh Chinese flowering cabbage leaves were placed on top of the plastic net, separated from the wet oviposition device below, thereby keeping the leaves fresh and promoting the health of the adults. The seedling paper was used to collect eggs that leaked through the net. The seedling paper absorbed water, becoming transparent and revealing the yellow pulp paper beneath, which facilitated the identification of eggs and larvae movement. **(2)** *Egg collecting and hatchery management*. After 1–2 days of oviposition, the seedling paper containing the eggs was transferred onto a new tray with layered two sheets of yellow pulp paper and a plastic grid to maintain humidity. Eggs were sprayed with 50 μg/mL natamycin to inhibit mold growth and incubated in an artificial climate chamber at 26 °C and 95% relative humidity (RH) and complete darkness until hatching. From early to later stages, eggs changed color from pale yellow to white. Just before hatching, two reddish-brown spots (mandibles) could be observed under a microscope. At the pre-hatching stage, the water in the tray was removed, and radish (*Raphanus sativus* L.) slices, sprayed with 200–400 μg/mL ciprofloxacin and 50 μg/mL natamycin (to inhibit bacterial and fungal growth), were placed around the eggs. **(3)** *Larval rearing*. After hatching, the color of the larvae changed from transparent white to grey-black, and they crawled to the underside of the radish slices to feed. The radish slices containing newly hatched larvae were transferred onto a tray covered with a layer of yellow pulp paper, allowing the slices to be stacked 3–5 layers deep. The larvae of the 1st and 2nd instars adopted a characteristic “C” shape, residing between the radish and the paper. Larvae preferred to bore into and eat the radish and crawl downward to molt. At the end of the 3rd instar, larvae gradually emerged from the inside of the radish, continued to eat a small amount on the surface of the radish, then ceased feeding and crawled downward. At this point, larvae need to be picked with a brush to the surface of a new paper towel to pupate.

Compared with the breeding device of Nagalingam et al. [19], the present breeding device has the following improvements: **(1)** *Device innovation*. The multi-layered egg-laying device (plastic net, seedling paper, yellow pulp paper, foam, sterile water tray) is designed to directly observe the activities of eggs and larvae through the transparent seedling paper, thereby improving the visibility of eggs and the collection efficiency. **(2)** *Standardization of egg incubation conditions*. This study standardized the incubation conditions (set at 26 °C, 95% RH, and a dark environment) and recorded the changes in egg color (light yellow → white → the appearance of reddish-brown spots) in detail to accurately determine the incubation stage. **(3)** *Management of the larval stage*. In this study, larvae were transferred by stacking radish slices in layers (3–5 layers) to facilitate observation of larval behavior and color changes of the larvae. After the eggs were transferred to rapeseed seedlings, Nagalingam et al. relied on natural hatching, and the larval management was relatively extensive.

For the larval bioassay, the 3rd instar fresh molting larvae of SFB were gently immersed in the conidia suspension of 1 × 10^8^ spores/mL (treatment group) or in 0.02% Tween 80 (control group) for 10 s and then placed on filter paper to dry. After drying, the larvae were transferred using a fine brush into Petri dishes containing radish slices pre-treated with 400 μg/mL ciprofloxacin to inibit bacterial growth. All dishes were maintained in an artificial climate chamber (LRH-250-Y, Guangdong Taihong Science Instrument Ltd., Shaoguan, China) at 26 ± 1 °C and 90 ± 5% RH. Radish slices were changed every 2–3 days. Each treatment and control consisted of 20 larvae, and each group was repeated three times. The mortality of the infected beetles was observed daily. An insect was considered dead if it did not move when its body (feet or antennae) was touched with a fine brush. The entire experiment was independently repeated twice.

### 2.3. Optimization of Bioassay System for SFB Larvae

#### 2.3.1. Effect of Plant Root Juice and Sulforaphane on EPF Conidia Germination

Plant root juice and sulforaphane: The roots of three plants (radish, pakchoi, and Chinese kale) were used in this study. The roots were thoroughly washed with water, and a juicer (Supor TJE06A, Zhejiang Supor Co., Ltd., Hangzhou, China) was employed to extract the crude juice. Then, the rude juice was filtered through gauze and a 0.22 μm filter membrane in turn to remove microbes. The filtered juice was diluted with sterile distilled water for subsequent experiments. Sulforaphane (Aladdin, Shanghai, China) was dissolved in sterile distilled water into the concentration of 500 mg/L used for the bioassay. Sulforaphane was set at a high concentration (500 mg/L) to demonstrate the positive effect of this compound on EPF.

Treatment: Each root juice and sulforaphane solution were respectively mixed in a 1:1 ratio with EPF conidial suspension (1 × 10^8^ spores/mL) and incubated statically for 1.5 h at 4 °C. Subsequently, the mixtures were inoculated onto PDA plates and cultured for 12 h at 26 °C. Finally, the germination rates of conidia were observed under a microscope. Each treatment was repeated three times, with a 0.02% Tween-80 solution set as the control. After 12 h, the germination of conidia was observed: the length of the germ tube exceeding 1/2 of the short diameter of the conidia was recorded as germination. Approximately 300 conidia were counted for each treatment at 40 × 10 magnification. Each treatment was conducted in triplicate, and the entire experiment was repeated twice; the conidia germination rate was calculated.

$$X=\frac{N1}{N}\times 100\%$$

where, *X* is the conidia germination rate (%); N1 is the number of germinated conidia (%); N is the total number of conidia observed.

#### 2.3.2. Bioassay of EPF on SFB Larvae Fed with Different Plant Roots

The method was the same as described above (Section 2.2). After treatment, the larvae were transferred into a Petri dish with the different plant roots (radish, pakchoi, and Chinese flowering cabbage). The results were checked as described in Section 2.2.

#### 2.3.3. Data Analysis

Corrected mortality was calculated according to Abbott formula:

$$Y=\frac{\left(T-CK\right)}{\left(1-CK\right)}\times 100\%$$

where, *Y* is the corrected mortality (%); T is the treatment mortality (%); CK is the control mortality (%).

The values of LT_50_ were evaluated based on Probit regression analysis. The DPS (Data Processing System, version 9.01) software was used to complete the statistical analyses [22]. When the *p*-value of the chi-square test is greater than 0.05, it means that there is no significant inconsistency between the data and the model, so the result is more reliable.

An ANOVA was performed using SPSS version 26.0 to test the significance. Data were compared with Duncan’s multiple range test and Tukey’s HSD test. Statistical significance was considered at *p* < 0.05.

## 3. Results

### 3.1. Bioactivity of EPF Strains to the SFB Adults and Larvae

**Figure 2:**
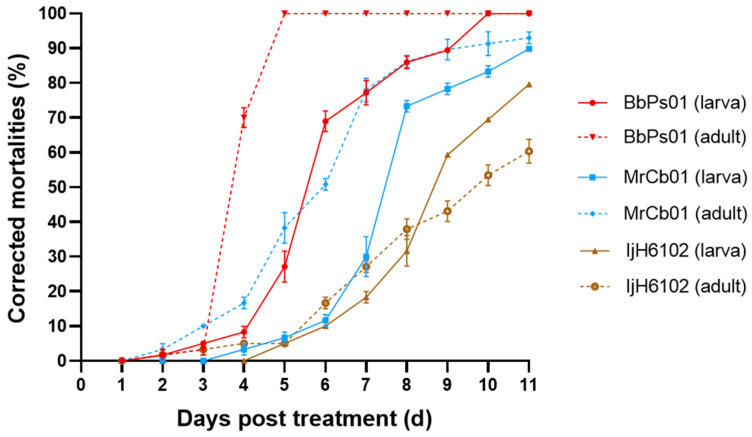
Mortality of SFB treated by EPF strains. Corrected mortality of the SFB adults infected by 1 × 10^8^ spores/mL conidial suspension of *B. bassiana* BbPs01, *M. robertsii* MrCb01, and *C. javanica* IjH6102. The SFB was treated by immersion of EPF conidial suspension at the concentration of 10^8^. There were 20 SFB in each treatment; the experiment was repeated three times. The solution of 0.02% Tween-80 was set as the control. SFB was reared using Chinese flowering cabbage.

For SFB adults, the results indicated that the mortality of SFB showed a time-dependent response to EPF at the concentration of 1 × 10^8^ spores/mL (Figure 2). In the earlier stages post-treatment (2–3 days post-treatment, dpt), all three EPF strains caused few SFB deaths. From 3–4 days post-treatment, BbPs01 induced a rapid increase in mortality, whereas MrCb01 and IjH6102 exhibited slower effects. By 5 dpt, BbPs01, MrCb01, and IjH6102 achieved SFB mortality rates of 100%, 38.33%, and 5.00%%, respectively. The LT-P equations and LT_50_ values were evaluated (Table 1). The LT_50_ values of BbPs01, MrCb01, and IjH6102 were 3.74, 5.52, and 9.38 d, respectively. It suggests that BbPs01 had the fastest and most potent effect on SFB adults, followed by MrCb01 and IjH6102 in turn.

For the larvae mortality, the results showed a dose/time-dependent response to EPF treatment (1 × 10^8^ spores/mL) with similar trends as the adults (Figure 2). The LT_50_ values of BbPs01, MrCb01, and IjH6102 were respectively evaluated as 5.65, 7.64, and 8.78 d (Table 1). Obviously, the larvae mortalities in the treatments of BbPs01 and MrCb01 were larger than the adult mortalities in the same strain treatment, which is an abnormal phenomenon because the mortality rate of larvae is usually higher than that of adult insects.

The adults or 3rd instar larvae were immersed in the EPF suspension of 1 × 10^8^ spores/mL for 10 s. The experiment was replicated three times, and 20 insects were used for each replicate. A solution of 0.02% Tween-80 was set as the control. SFB was reared using Chinese flowering cabbage. DPS version 9.01 was used for time-dose mortality model analysis. 

### 3.2. Optimization of Bioassay System of EPF Larvae on P. Striolata

#### 3.2.1. Effect of Root Juices and Sulforaphane on EPF Conidial Germination

**Figure 3:**
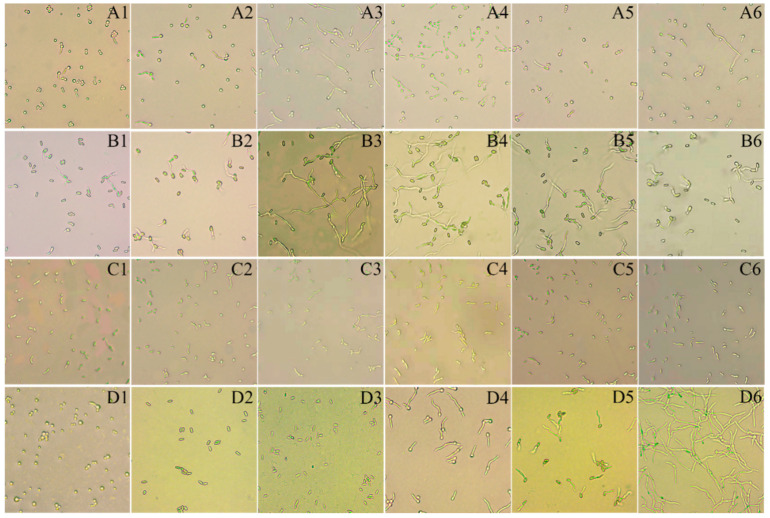
Effect of EPF conidial germination under different treatments of plant juices. (**A1**–**A6**), *B. bassiana* BbPs01; (**B1**–**B6**), *M. robertsii* MrCb01; (**C1**–**C6**), *C. javanica* IjH6102; where 1/2/3/4/5/6 indicates the juice treatments of radish/radish 1/3×/pakchoi 1/3×/Chinese flowering cabbage 1/3×/Chinese kale 1/3×/cabbage 1/3×. 1/3× means the concentration of the host plant root has been diluted three times, not the concentration of the spore suspension 1 × 10^8^ has been diluted three times. The lowest conidial germination was observed under the microscope in the treatments of radish juice. (**D1**–**D3**)**,** sulforaphan (500 mg/L) to BbPs01/MrCb01/IjH6102; (**D4**–**D6**), control (0.02% Tween-80) for BbPs01/MrCb01/IjH6102. Under the microscope, the sulforaphane treatment group had apparent inhibitory effect compared with the control group. Determination of conidia germination rate: Conidia germination was observed after 12 h, the length of the germ tube exceeding 1/2 of the short diameter of the conidia was recorded as germination. Approximately 300 conidia were counted for each treatment at 40 × 10 magnification.

**Figure 4:**
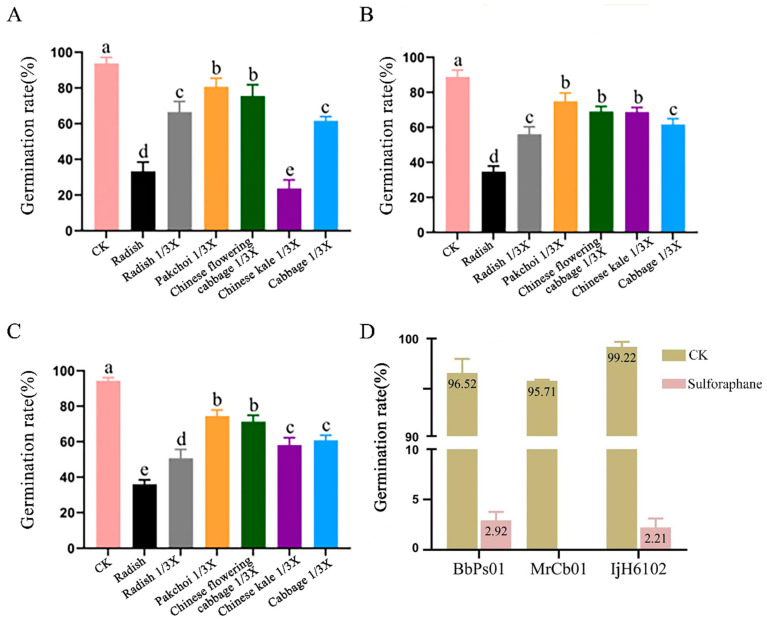
Effect of host plant root juices and sulforaphane on EPF conidial germination. (**A**–**D**) The data analysis of BbPs01/MrCb01/IjH6102/sulforaphane (500 mg/L). 1/3× means the concentration of the host plant root has been diluted three times, not the concentration of the spore suspension 1 × 10^8^ has been diluted three times. The root juices and sulforaphane were respectively mixed 1:1 with EPF conidial suspension of 1 × 10^8^ spores/mL and static cultivation for 1.5 h at 4 °C. Then, they were inoculated on PDA plate to further culture for 12 h at 26 °C for the germination rates check. The experiment was repeated three times; the control was 0.02% Tween-80 solution. Determination of conidia germination rate: Conidia germination was observed after 12 h. The length of the germ tube exceeding 1/2 of the short diameter of the conidia was recorded as germination, and the number of conidia germinated was counted under a microscope at 40 × 10 magnification. About 300 conidia were counted for each treatment. The different letters on columns indicate the significant difference (*p* < 0.05) by Tukey’s HSD test.

The results indicated that the conidial germination rate of the EPF strains was affected to varying degrees by the treatments. Totally, pakchoi and Chinese flowering cabbage juices had little side effects on the three EPF strains, BbPs01, MrCb01, and IjH6102, but radish, Chinese kale, cabbage, and sulforaphane had large impacts on conidial germination after 24 h of treatment (Figure 3 and Figure 4).

#### 3.2.2. Bioactivity of EPF on SFB Larvae Fed by Different Plant Roots

**Figure 5:**
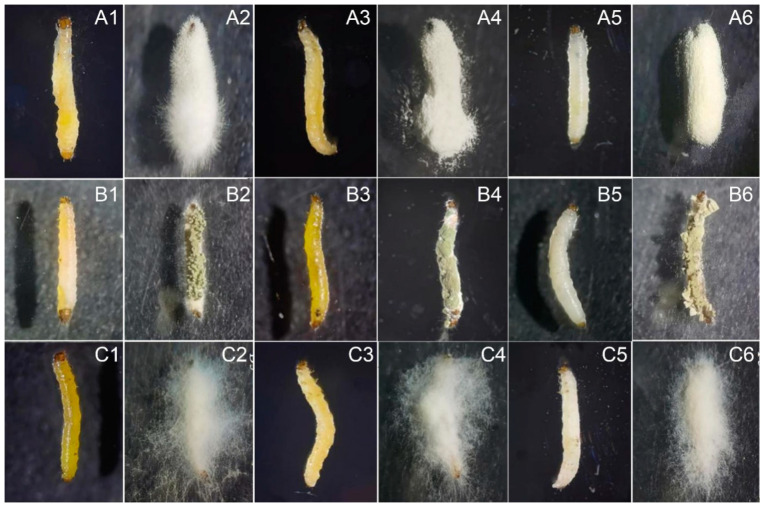
Symptoms of SFB larvae treated by EPF and fed on different plant roots. (**A1**–**C6**), treatment of BbPs01/MrCb01/IjH6102. **1/2**, just-dead larvae/zombie worms fed on Chinese flowering cabbage; **3/4**, just-dead larvae/zombie worms fed on pakchoi; **5/6**, just-dead larvae/zombie worms fed on radish. Just-dead and zombie worms were photographed 7 and 11 days after treatment, respectively.

The just-dead larvae fed on different roots exhibited different colors after EPF treatment (Figure 5). Larvae reared on pakchoi and Chinese flowering cabbage displayed yellow bodies, while those feeding on radish showed white bodies. This variation in coloration may be attributed to the presence of plant-derived pigments in pakchoi and Chinese flowering cabbage, which are likely absent in radish.

**Figure 6:**
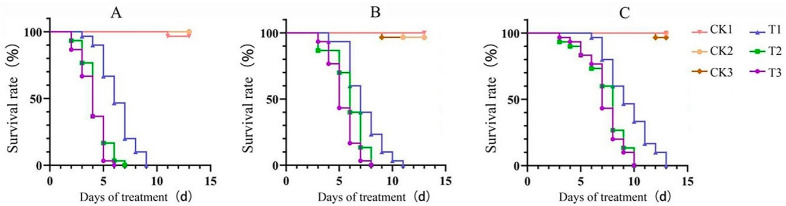
Bioactivity of EPF on SFB larvae fed with different cruciferous plants. (**A**–**C**) The treatment of BbPs01/MrCb01/IjH6102. **T1/2/3** is respectively fed by the roots of radish/pakchoi/Chinese flowering cabbage with the EPF treatment; **CK1/2/3** is only fed by radish/pakchoi/Chinese flowering cabbage, with no EPF treatment. The immersion method was used to bioassay. The 3rd instar larvae were immersed in the EPF suspension of 1 × 10^8^ spores/mL for 10 s, then they were introduced onto the fresh plant roots and cultured. Each treatment and control consisted of 20 larvae, and each group was repeated three times. The results were checked each day, and the insects that did not move after having their bodies touched with a hair brush were accepted as dead.

The results also indicated that feeding with different roots closely influenced the survival rates of larvae treated with EPF (Figure 6). Totally, larvae fed on radish root exhibited higher survival rates compared to those fed on the roots of pakchoi and Chinese flowering cabbage, which suggested that radish has more apparent impacts on the three EPF strains, thereby reducing their bioactivity against SFB larvae.

Subsequently, the LT-P equations and LT_50_ values of EPF strains on larvae fed different roots were evaluated (Table 2). The results indicated that feeding radish to the larvae gave the strain BbPs01/MrCb01/IjH6102 the LT_50_ values of 5.67/6.50/8.72 d, while feeding Chinese flowering cabbage resulted in the lowest LT_50_ values of 3.02/4.53/6.61 d. Obviously, feeding larvae radish had a larger impact on EPF activity. These results suggested that the larvae bioassay system should be optimized by rearing the insects with Chinese flowering cabbage.

The 2nd instars were immersed in the EPF suspension of 1 × 10^8^ spores/mL for 10 s, and 20 larvae were used for each replicate. SFB was reared using radish, pakchoi, and Chinese flowering cabbage. LT-P equations and LT_50_ values of EPF strains were evaluated for larvae reared with different root systems. 

## 4. Discussion

SFB larvae are soil-dwelling, and most EPF are also soil-inhabiting fungi. Therefore, making larvae bioassays a more realistic approach for screening active fungal strains. Currently, EPF-based control strategies for SFB in the field primarily involve the development of various formulations—such as water-based, oil-based, and nano/microencapsulated forms—or the application of CRISPR-edited strains to enhance fungal viability and spore germination under field conditions [23,24]. These formulations are typically applied through spraying or broadcasting, enabling contact between the EPF and the target pest. However, the difficulty in acquiring and rearing SFB larvae remains a major constraint, limiting the development and application of mycoinsecticides in IPM programs for SFB. To date, no EPF-based mycoinsecticides have been officially registered for SFB control in China. Although Nagalingam and Costamagna reported the rearing methods for SFB, the techniques for larvae bioassay are not enough. Undoubtedly, this research provides a good method for screening active fungal strains in SFB.

Obviously, the different efficacy of EPF strains against SFB is not only due to fungal genetic features but also to environmental factors that closely influence the interactions of plant-insect-EPF. Cruciferous plants produce a wide array of secondary metabolites with multiple functions, such as defense against insects and pathogens. Through long-term co-evolution, SFB has developed adaptations to the unique glucosinolate- myrosinase defense system of crucifers, commonly referred to as the “mustard oil bomb”. SFB possesses highly active endogenous myrosinase enzymes, which hydrolyzes with at least fourfold higher efficiency than aromatic and indolic glucosinolates and beta-O-glucosides [25,26,27]. Glucosinolates themselves are non-toxic compounds stored in the vacuoles of plant cells. However, when plant tissues are damaged or eaten by insects, myrosinase comes into contact with glucosinolate, which hydrolyzes to form toxic compounds such as isothiocyanates, thiocyanates, or nitriles, all of which are toxic to insects and microorganisms [28,29,30,31]. Importantly, EPFs have not co-evolved to the “mustard oil bomb” yet; because EPF are not the pathogens of cruciferous plants, there are also few reports about EPF being endophytic in cruciferous plants. When SFB feeds on host plants and triggers “mustard oil bomb” exploding, the resulting chemical reaction may negatively impact EPF conidia present on the insect cuticle or plant surface. This interaction could partially explain the reduced efficacy of EPF-based control strategies against SFB in cruciferous crop systems.

In our experiment on larvae rearing, we observed that radish causes significant damage to EPF compared to pakchoi and Chinese flowering cabbage; the results were supported by the plant root juice tests, which showed that radish juices and sulforaphane are more toxic to EPF. This differential impact can be attributed to variations in the “mustard oil bomb” system across different plant species. Specifically, compared with pakchoi and Chinese flowering cabbage, radish contains higher glucosinolate, which is hydrolyzed into sulforaphane by myrosinase [21,32]. The optimization of the bioassay system for EPF against SFB larvae hinges on creating a balance between co-culturing the larvae and EPF in a way that minimizes damage to both organisms. This research just provides a choice to solve the problems. However, certain limitations remain. The plant sap assays cannot fully capture the physiological and biochemical responses of SFB when interacting with plants, and the concentration of plant defense compounds within the insect may also influence fungal infection rates. These aspects warrant further investigation through additional experimental work.

In conclusion, this research provided a bioassay system for screening EPF strains active against SFB. Our results indicate that larvae fed with Chinese flowering cabbage roots are the best choice for the bioassay of EPF against SFB larvae, while plants such as radish, rich in sulforaphane, severely impair EPF germination rates and compromise the accuracy of the bioassay. These findings provide valuable insights for the development of mycoinsecticides and the sustainable control of SFB.

## Figures and Tables

**Table 1:** LT-P equation and LT_50_ of EPF against SFB.

EPF Strain	LT-P Equation (y = A + Bx) and Significant Test							LT_50_ (95% Confidence Interval, ×10^6^ Spores/mL)		SFB
	Intercept (A)	Slope (B)	SE	R	χ^2^	DF	*p* *			
BbPs01	−6.4358	19.9537	2.5585	0.9795	0.5304	1	0.4664	3.74	(3.60–3.87)	adults
MrCb01	0.7881	5.6747	0.4247	0.9871	5.9961	7	0.5402	5.52	(5.20–5.83)	
IjH6102	−0.0238	5.1670	0.6452	0.9872	1.566	5	0.9053	9.38	(8.78–10.27)	
BbPs01	−1.6211	8.8061	0.7288	0.9175	5.8315	5	0.3230	5.65	(5.35–5.91)	larvae
MrCb01	−3.4999	9.6228	0.9387	0.9641	9.9295	4	0.0416	7.64	(7.32–7.93)	
IjH6102	−3.7913	9.3184	1.7005	0.9795	1.3739	2	0.5031	8.78	(8.24–9.16)	

* if *p* ≥ 0.05, indicating the model credible.

**Table 2:** LT-P equation and LT_50_ of EPF against SFB larvae reared with different plant roots.

Treatment	LT-P Equation (y = A + Bx) and Significant Test							LT_50_ (95% Confidence Interval, d)
	Intercept (A)	Slope (B)	SE	R	χ^2^	DF	*p* *	
BbPs01								
radish	−1.2612	8.3083	0.7686	0.9975	1.0016	4	0.9096	5.67 (5.42–5.96)
pakchoi	1.1145	6.8293	0.6669	0.9800	5.2306	3	0.1557	3.71 (3.49–3.93)
Chinese flowering cabbage	1.2657	7.7841	0.8072	0.9928	7.0994	3	0.0688	3.02 (2.84–3.25)
MrCb01								
radish	−2.2915	8.9729	1.1702	0.9946	0.6289	3	0.8898	6.50 (5.98–6.86)
pakchoi	−4.9387	12.2585	1.1733	0.8976	5.8392	3	0.1197	6.47 (6.23–6.69)
Chinese flowering cabbage	−2.0057	10.6790	0.9995	0.9647	9.7197	4	0.0454	4.53 (4.34–4.75)
IjH6102								
radish	−3.3354	8.8608	0.9731	0.9952	1.0100	4	0.9083	8.72 (8.34–9.06)
pakchoi	−2.0050	8.3098	0.9637	0.9708	5.4900	3	0.1392	6.97 (6.67–7.29)
Chinese flowering cabbage	−1.9607	8.4843	0.7950	0.9762	7.6721	4	0.1044	6.61 (6.34–6.92)

* if *p* ≥ 0.05, indicating the model credible.

## Data Availability

The original contributions presented in this study are included in the article. Further inquiries can be directed to the corresponding author.
